# 64Cu-ATSM Hypoxia Positron Emission Tomography for Detection of Conduit Ischemia in an Experimental Rat Esophagectomy Model

**DOI:** 10.1371/journal.pone.0131083

**Published:** 2015-06-22

**Authors:** Seong Yong Park, Won Jun Kang, Arthur Cho, Ju Ri Chae, Ye Lim Cho, Jung Young Kim, Ji Woong Lee, Kyung Young Chung

**Affiliations:** 1 Department of Thoracic and Cardiovascular Surgery, Yonsei University, College of Medicine, Seoul, Republic of Korea; 2 Department of Thoracic and Cardiovascular Surgery, Ajou University, School of Medicine, Suwon, Republic of Korea; 3 Department of Nuclear Medicine, Yonsei University, College of Medicine, Seoul, Republic of Korea; 4 Molecular Imaging Research Center, Korea Institute of Radiological and Medical Sciences, Seoul, Republic of Korea; Baylor College of Medicine, UNITED STATES

## Abstract

**Background:**

We designed a hypoxia-imaging modality to detect ischemia of the gastric conduit after esophagectomy.

**Materials and Methods:**

A rat esophagectomy model was created using 12-16-week-old, 300-350 g male Sprague-Dawley rats. In the operation group (n=6), partial gastric devascularization was performed by ligating the left gastric artery and the short gastric arteries and an esophagogastric anastomosis was performed. In the control group (n=6), the esophageal-gastric junction was incised and suturing was performed without gastric devascularization. Positron emission tomography (PET) images were taken using a microPET rodent model scanner, 24 h after the initial operation, after injection of 200 μCi 64Cu-diacetyl-bis (N4-methylsemicarbazone) (64Cu-ATSM) and pimonidazole 120 mg/kg. After microPET imaging, autoradiography and immunohistochemistry were performed.

**Results:**

The PET image revealed 64Cu-ATSM uptake at the fundus in the operation group 3 h after 64Cu-ATSM injection. The maximum percentage of the injected dose per gram of tissue was higher in the operation group (0.047±0.015 vs. 0.026±0.006, *p*=0.021). The fundus/liver ratio was also higher in the operation group (0.541±0.126 vs. 0.278±0.049, *p*=0.002). Upon autoradiography, 64Cu-ATSM uptake was observed in the fundus in the operation group, and was well-correlated to that observed on the PET image. Upon immunohistochemistry, expression of hypoxia-inducible factor 1a and pimonidazole were significantly increased at the fundus and lesser curvature compared to the greater curvature in the operation group.

**Conclusion:**

Hypoxia PET imaging with 64Cu-ATSM can detect ischemia in a rat esophagectomy model. Further clinical studies are needed to verify whether hypoxia imaging may be useful in humans.

## Introduction

Esophagectomy is the treatment of choice for early and locally advanced esophageal cancer. After esophagectomy, esophageal reconstruction is generally performed via gastric pull-up and esophagogastrostomy [1]; however, an esophagogastric anastomotic leakage develops in 5–20% of all esophagectomy cases [1,2]. The operative mortalities of esophagectomy for esophageal cancer has been reported up to 10% [1], and between 30% and 50% of these deaths are related to anastomotic leakage [3]. Therefore, the elimination of anastomotic leakages is essential for improving the morbidity and mortality after esophagectomy. Several factors are linked to development of anastomosis leakage; ischemia of the gastric conduit is a major factor [3]. Additionally, clinical detection and measurement of ischemia of the gastric conduit during the postoperative period is difficult. Oezcelik et al. recently reported that chest computed tomography (CT) was not useful for detecting conduit ischemia or anastomosis breakdown, and that endoscopy was more valuable than chest CT for detecting ischemia [4]. However, endoscopy is invasive and can damage anastomoses; moreover, grading of gastric mucosal ischemia by an endoscopist can be subjective.

Currently, nuclear imaging techniques, particularly positron emission tomography (PET), are best when used to detect and assess tissue hypoxia because of the availability of several radiotracers that are selectively entrapped within regions of hypoxic tissue [5]. Hypoxia PET imaging has been applied to cerebral stroke and cancer imaging. We hypothesized that hypoxia PET imaging would detect ischemic areas of the gastric conduit after esophagectomy and esophagogastrostomy, because ischemia would be indicated by hypoxia of a particular tissue or organ. Accordingly, we performed an animal study to verify whether hypoxia PET imaging could detect ischemia of a gastric conduit.

## Materials and Methods

### Operation

**Fig 1:**
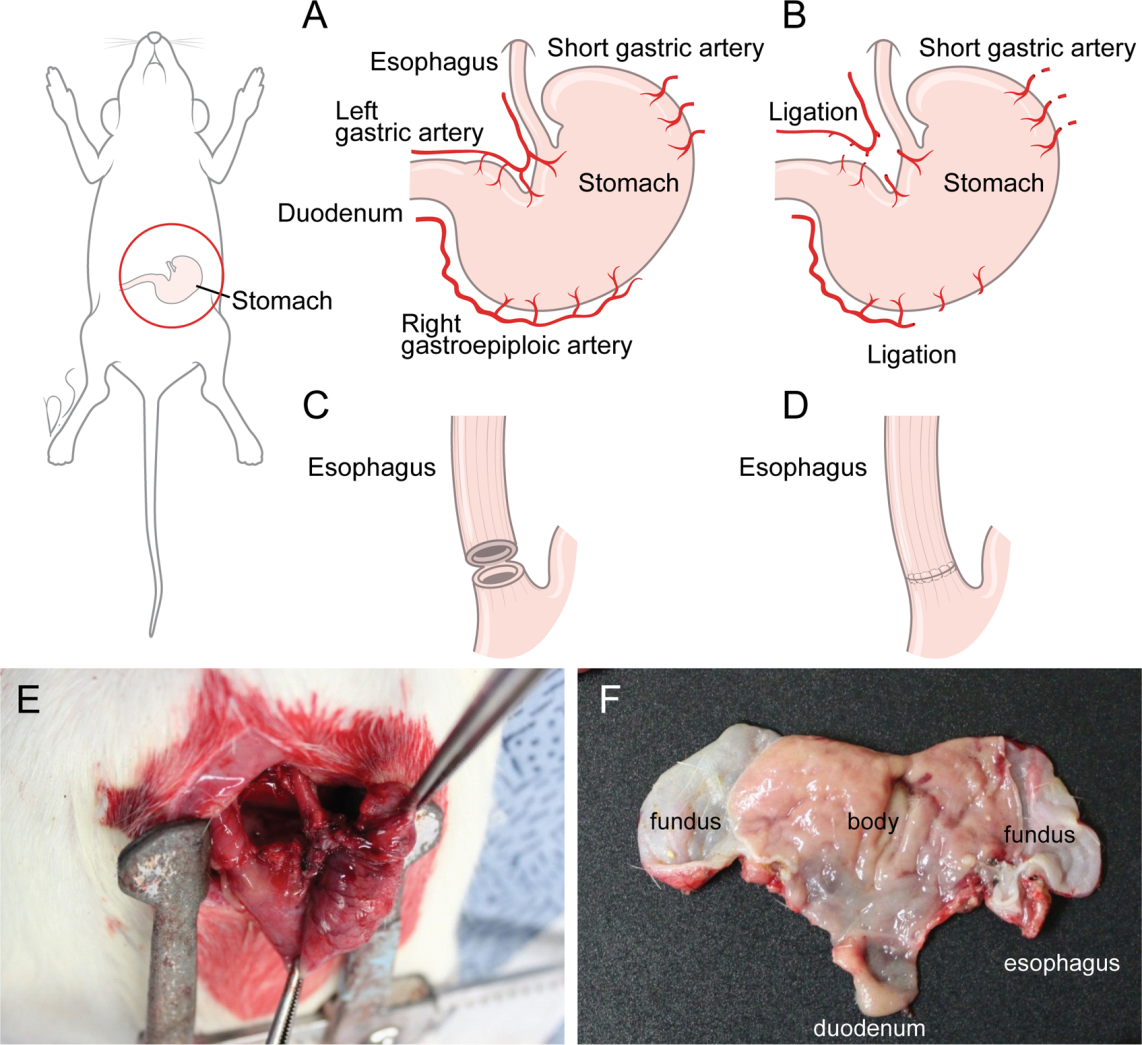
Operative view. A. Normal vascular anatomoy of rat. B. Partial devascularization was done by ligating the left gastric artery and short gastric arteries. C. The esophageal-gastric junction was then incised around 50% of the circumference, leaving the small bridge of tissue at posterior part of esophagogastric junction. D. The esophagogastric anastomosis was sutured with interrupted 5–0 polypropylene sutures. E. Operative pictures after all procedures in operation group. F. After microPET imaging, the stomach was incised along the lesser curvature to obtain autoradiographic images.

The current study was approved by the institutional Animal Care and Use Committee (No. 2013–0350) of Yonsei University, College of Medicine. Based on previous studies, a rat esophagectomy model was created using 12-16-week old, 300-350-g, male Sprague-Dawley rats [6,7]. Twelve rats were housed (3 per cage) in conventional suspension cages, and given food and water ad libitum until the time of surgery. Six rats were each randomly assigned to the control (n = 6) and the operation groups (n = 6). In all rats, a 3-cm median laparotomy incision was made under inhaled sevoflurane anesthesia using a rodent ventilator. In the operation group, partial gastric devascularization was performed by ligating the left gastric artery and the short gastric arteries (Fig 1A and 1B). The esophageal-gastric junction was then incised around 50% of the circumference. This left a small bridge of tissue at posterior part of esophagogastric junction for simplifying the anastomotic suturing. The esophagogastric anastomosis was sutured with interrupted 5–0 polypropylene sutures (Fig 1C, 1D and 1E). All laparotomy incisions were closed with continuous 3–0 silk sutures. Animals were allowed free access to water only after operation. The control group underwent only incision of the esophageal-gastric junction; suturing was performed without partial gastric devascularization.

### MicroPET scan

64Cu-diacetyl-bis (N4-methylsemicarbazone) (64Cu-ATSM) was obtained from Korean Cancer Center. PET imaging was performed using a microPET rodent scanner (Siemans Inveon MicroPET) 24 h after the initial operation. Before PET imaging, water and food were permitted for 12 hours. After fasting, the animals were injected with 200 μCi 64Cu-ATSM via the tail vein and 120 mg/kg pimonidazole intraperitoneally. Each rat was placed near the center of the field of view of the microPET 3 h after 64Cu-ATSM injection, where the highest image resolution and sensitivity were available. Static imaging was performed for 20 min at 3 h after injection of ATSM. A region of interest (ROI) was drawn in the gastric fundus and a dose of 64Cu-ATSM semiquantitated to the “max percent” value was injected per gram of tissue (%ID/g). The fundus/liver ratio was calculated by dividing the %ID/g of the fundus by the %ID/g of the liver. A reference region of the liver was drawn in the right hepatic lobe.

### Autoradiography

Immediately after microPET imaging, the rats were euthanized and the stomachs excised along the lesser curvature (Fig 1F). Excised stomachs were transferred to a chilled autoradiography cassette and stored for 12 h at -4°C. Screens were read using an FLA7000 scanner (Fujifilm, Tokyo, Japan). ROIs were selected on the greater curvature of the stomach where the blood supply was intact, and in the fundus where the blood supply was not intact because of partial devascularization of the stomach. The optical densities of autoradiographic signals were measured using Multi Gauge 3.2 software (Fujifilm, Tokyo, Japan). Autoradiographic images and ROIs were compared between the two groups.

### Histological evaluation and immunohistochemistry

After autoradiography, stomach tissue was fixed in 2% (v/v) formalin, embedded in paraffin for 24 h, sectioned at 5-μm thickness, and stained with hematoxylin and eosin (H&E). Four sections were prepared from each rat; two from the great curvature of the stomach (where the blood supply was intact) and two from the fundus where the blood supply was not intact because of partial devascularization of the stomach. These sections were from the same ROIs evaluated via autoradiography. From the 12 rats, a total of 48 sections was prepared. Each slide was stained with hypoxia-inducible factor 1a (HIF-1a) antibody and hypoxyprobe-1 anti-pimonidazole mouse monoclonal IgG1 antibody (MAb1). The percentage positivies for MAb1 and HIF-1a were quantified using ImageJ 1.41o software (National Institutes of Health) and compared between the two groups.

### Statistical analysis

All parameters were described as mean ± standard deviation of mean for continuous variables. Statistical analyses were performed using a non-parametric Mann-Whitney U-test to evaluate the significance of differences in values between different areas. Pearson correlation test was performed to verify the relationships between density of immunohistochemistry stain and PET uptake. A P-value of <0.05 was considered to indicate a statistically significant difference. All statistical procedures were performed using SPSS software (version 20.0; SPSS Inc., Chicago, IL, US).

## Results

### MicroPET imaging

**Fig 2:**
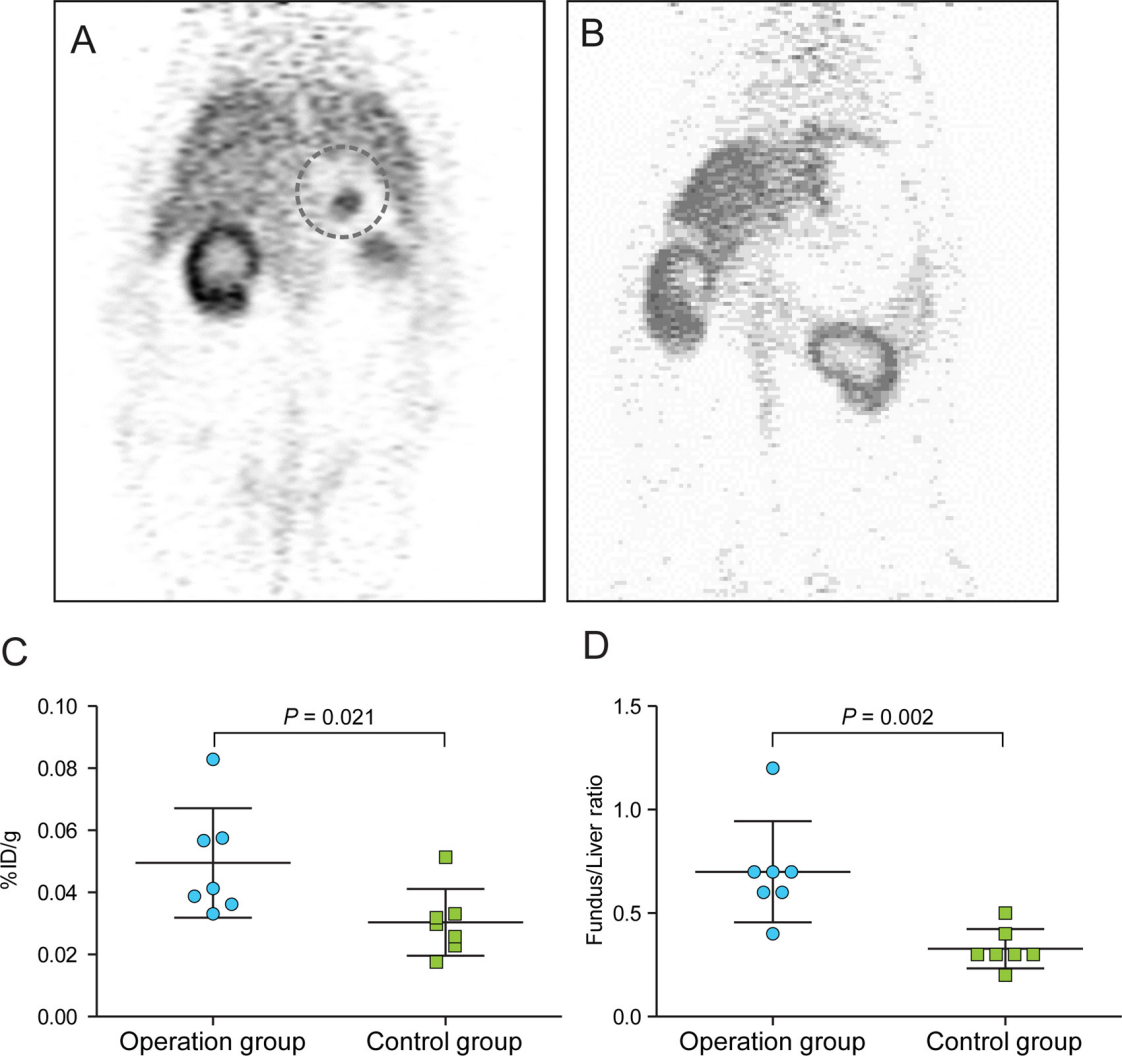
MicroPET imaging. A. Operation group. 64Cu-ATSM uptake was observed in the fundus (dotted line). B. Control group. 64Cu-ATSM uptake was not observed in the fundus. C. Comparison of %ID/g values between the operation and control groups. D. Comparison of the fundus/liver ratio (%ID/g of fundus area by %ID/g of liver) between the operation and control groups.

On static PET imaging, Cu-ATSM uptake at the fundus was observed in the operation group on the 3-h PET image (Fig 2A), but such abnormal 64Cu-ATSM uptake was not evident in the control group (Fig 2B). The mean %ID/g of the fundus in the operation group was 0.047 ± 0.015. In the control group, the area corresponding to the fundus was chosen as an ROI, because there was no definite area of uptake in the control group, and the mean %ID/g of this area was 0.026 ± 0.006; the difference was significant (Mann-Whitney test, *p* = 0.021, Fig 2C). The fundus/liver ratios were 0.541 ± 0.126 and 0.278 ± 0.049, respectively (operation group and control group, Mann-Whitney test, *p* = 0.002, Fig 2D).

### Autoradiography

**Fig 3:**
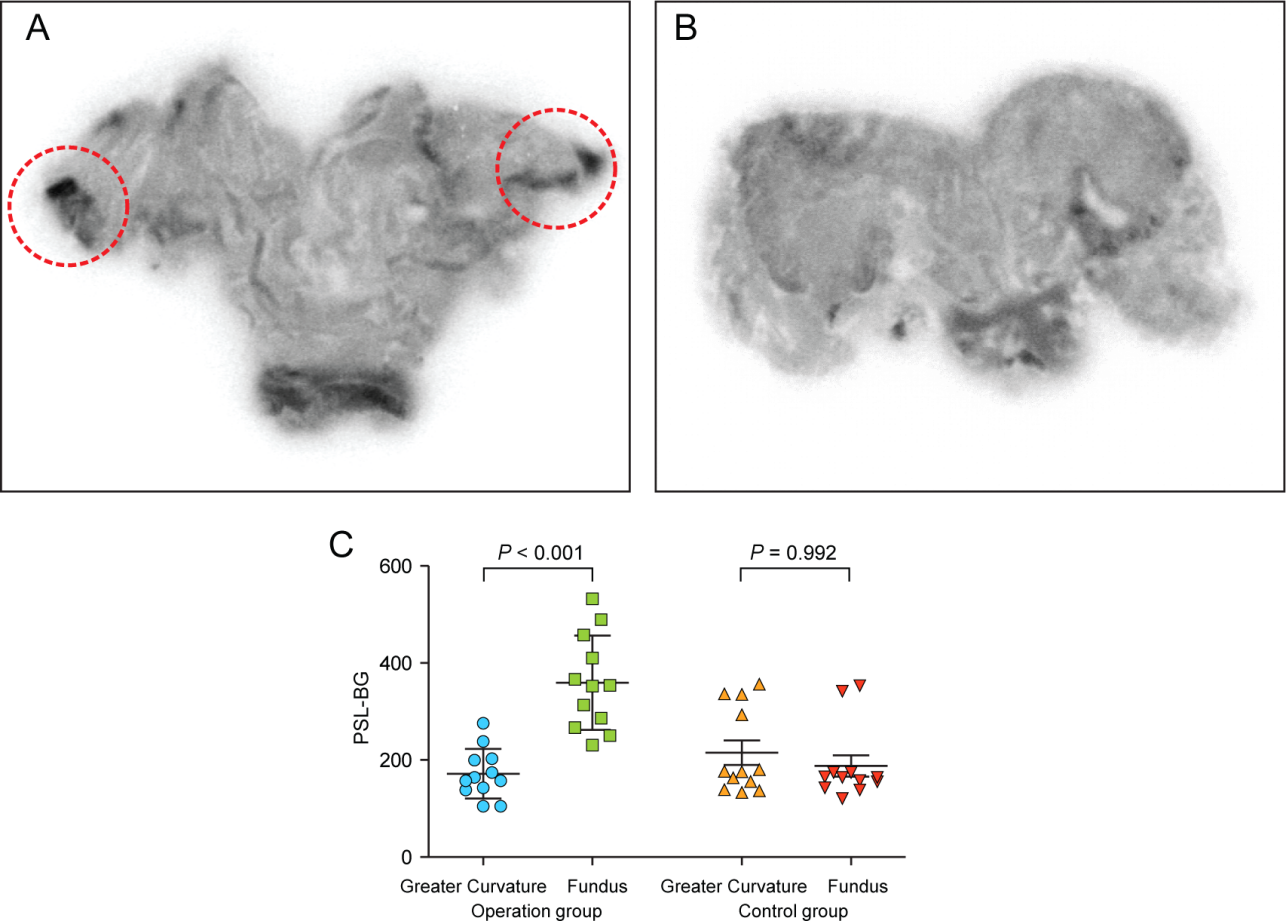
Autoradiography. A. Operation group. 64Cu-ATSM uptake was observed in the fundus. B. Control group. No abnormal uptake was observed. C. The intensities of 64Cu-ATSM uptake in the control and operation groups. 64Cu-ATSM uptake was two-fold higher in the fundus compared to the greater curvature in the operation group (179.8120 ± 50.6658 vs. 353.3640 ± 85.0633, Mann-Whitney test, p<0.001), but uptake was similar in the fundus and greater curvature of the control group.

Autoradiographic images are shown in Fig 3. The principal region of 64Cu-ATSM uptake was the fundus in the operation group (Fig 3A). In the control group, no definite 64Cu-ATSM uptake by stomach tissue was evident (Fig 3B). The intensities of 64Cu-ATSM uptake are compared in Fig 3C; 64Cu-ATSM uptake was two-fold higher in the fundus than the greater curvature in the operation group (179.812 ± 50.665 PSL-BG vs. 353.364 ± 85.063 PSL-BG, Mann-Whitney test, p<0.001), but, in the control group, 64Cu-ATSM uptake was similar in the fundus and greater curvature.

### Immunohistochemistry

**Fig 4:**
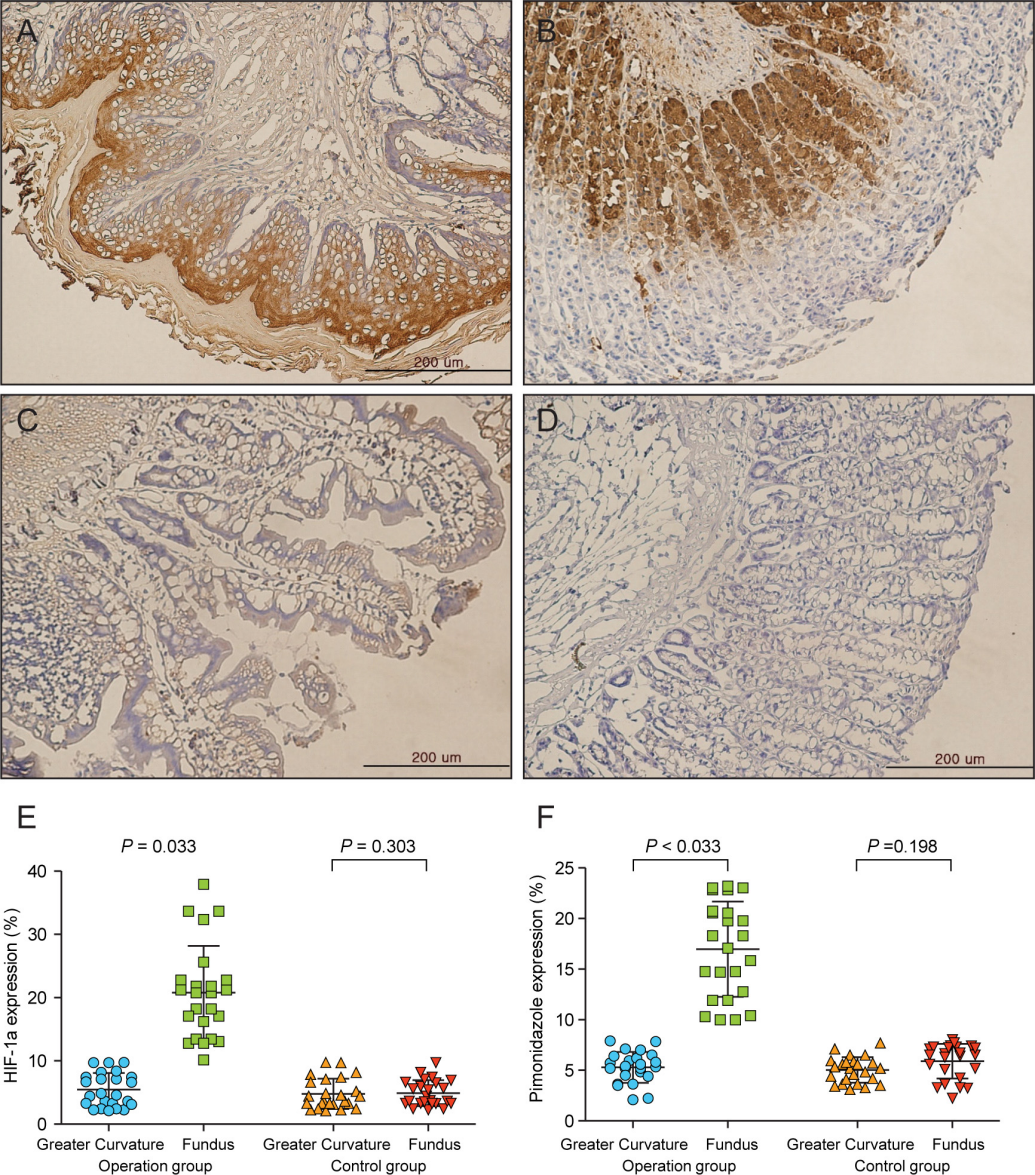
Immunohistochemistry. A. HIF-1a expression in the fundus of the operation group. B. Pimonidazole expression in the fundus of the control group. Pimonidazole and HIF-1a were expressed in the fundus. C. HIF-1a expression in the fundus of the control group. D. Pimonidazole expression in the fundus of the control group. Pimonidazole and HIF-1a were not expressed in the fundus. E. Comparison of HIF-1a expression levels between the fundus and greater curvature in each group. F. Comparison of pimonidazole expression levels between the fundus and greater curvature in each group. HIF-1a and pimonidazole expression was significantly higher in the fundus than the greater curvature in the operation group.

The expression levels of pimonidazole and HIF-1a in the fundus and greater curvature were compared. In the operation group, pimonidazole and HIF-1a were expressed in the fundus but not the greater curvature (Fig 4A and 4B). In the control group, pimonidazole and HIF-1a expression was observed in neither the fundus nor the greater curvature (Fig 4C and 4D). In the operation group, pimonidazole 1 and HIF-1a expression was significantly higher in the fundus than the greater curvature (Fig 4E and 4F).

**Fig 5:**
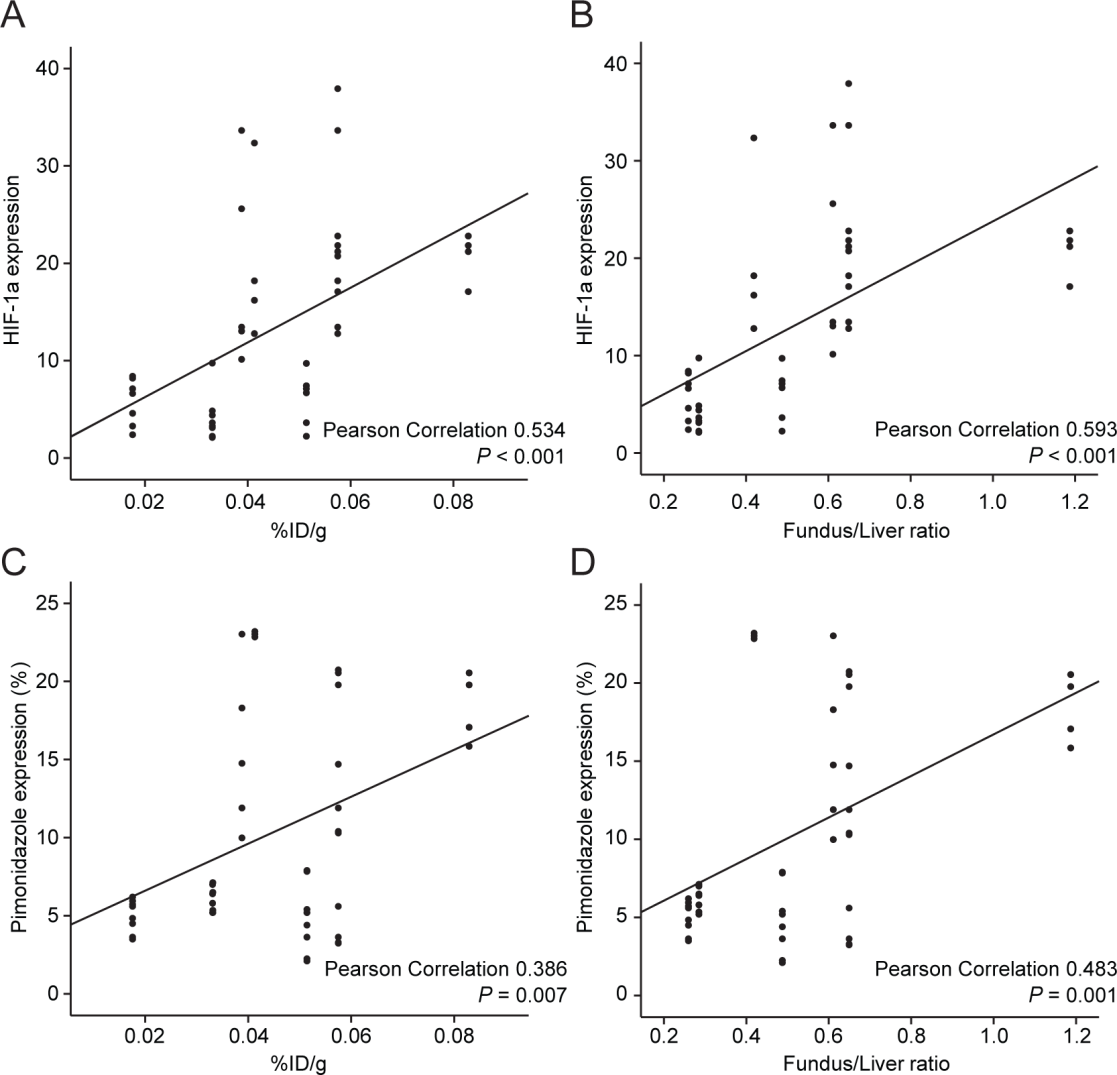
The correlations between expression of immunohistochemistry and PET parameters. A. HIF-1a expression and %ID/g (Pearson correlation 0.534, *p*<0.001). B. HIF-1a expression and Fundus/Liver ratio (Pearson correlation 0.593, *p*<0.001). C. Pimonidazole expression and %ID/g (Pearson correlation 0.386, *p* = 0.007). D. Pimonidazole expression and Fundus/Liver ratio (Pearson correlation 0.483, *p* = 0.001).

The correlations between expression of immunohistochemistry and PET parameters were analysed. HIF-1a expression was correlated with both %ID/g and Fundus/Liver ratio (Pearson correlation 0.534, *p*<0.001 and Pearson correlation 0.593, *p*<0.001, respectively. Fig 5A and 5B). Pimonidazole expression was also correlated with both %ID/g and Fundus/Liver ratio (Pearson correlation 0.386, *p* = 0.007 and Pearson correlation 0.483, *p* = 0.001, respectively. Fig 5C and 5D).

## Discussion

After esophagectomy and gastric reconstruction, anastomotic leakage develops in about 5–20% of patients. Ischemia of the gastric conduit is a major cause of this problem [2]. Preparation of the stomach for gastric pull-up requires ligation of the left gastric, left gastroepiploic, and short gastric arteries. After preparation of gastric conduit, the blood supply to fundus of stomach is derived from right gastroepiploic artery arcade and communicated rich submucosal plexus of vessels [8]. Although frank gastric necrosis is rare if the stomach is properly prepared [9], unexpected ischemia of the gastric fundus often develops [8,10]. Additionally, clinical detection and measurement of ischemia of the gastric conduit during the postoperative period is difficult. Chest CT is not useful and endoscopy is both subjective and invasive. Detecting and measuring ischemia of the gastric conduit in the postoperative period via a non-invasive imaging modality is essential to allow of decision-making in difficult clinical situations. After esophagectomy and gastric reconstruction, if ischemia of the gastric conduit is severe, take-down of the gastric conduit should be considered to avoid fulminant necrosis of the conduit and resulting sepsis. If ischemia is both mild and not extensive, conservative management can be considered.

We hypothesized that hypoxia PET imaging would detect conduit ischemia. In fact, ischemia and hypoxia are different phenomena: ischemia refers to low blood circulation in a particular tissue or cell, whereas hypoxia refers to low-level oxygen saturation in a particular tissue or cell. Nevertheless, hypoxia was generally present in ischemic areas in several previous studies. Some researchers have reported that HIF-1a is expressed in ileal mucosa cells after superior mesenteric artery occlusion or hemorrhagic shock (ischemia) in rats [11]. Clinically, hypoxia imaging has been applied to study acute cerebral ischemia, using hypoxia-detecting agents such as 18F-fluoromisonidazole [12]. Although ischemia and hypoxia differ, they seem to share a common pathophysiology. Based on these observations, we attempted to use hypoxia PET imaging to detect ischemia of a gastric conduit, using the rat esophagectomy model described on previous studies [6,7]. As esophagectomy and reconstruction are very invasive and associated with high mortality, intrathoracic esophagectomy with anastomosis is difficult to perform in animals. Fortunately, the intraabdominal esophagus of the rat is relatively long, and previous investigators successfully performed partial resection of this section of the esophagus, and intraabdominal anastomosis, in the rat [6,7]. Also, the vascular anatomy of the rat stomach is quite similar to that of the human. This animal model seeks to mimic gastric ischemia, not intrathoracic esophagectomy and/or gastric pull-up. We performed 20 rat esophagectomies prior to initiation of main experiment.

We used 64Cu-ATSM as a radiotracer for hypoxia imaging in this study with two reasons. First, 64Cu-ATSM is very lipophilic with low molecular weight, therefore is more permeable to high cell membrane than other imidazole-ring group hypoxia agents such as 18F-fluoromisonidazole (fMISO). 64Cu-ATSM can permeate the cell membrane freely and converted from 64Cu^2+-^ATSM to 64Cu^1+^-ATSM in cell. 64Cu^1+^-ATSM cannot permeate the cell membrane and deposit in the cell. To convert from 64Cu^1+^-ATSM to 64Cu^2+^-ATSM needs normoxia status. [13,14] Second, Cu-ATSM has been studied not only in tumor conditions but also in non-tumor conditions such as myocardial perfusion and cerebral ischemia, in contrast to other imidazole-ring group hypoxia tracers which has been studied mainly in tumor conditions [15–17]. On 64Cu-ATSM PET imaging, radiotracer uptake was observed in the fundus of the operation group. The gastric fundus is the area most susceptible to ischemia after ligation of the left gastric and short gastric arteries. The area of 64Cu-ATSM uptake on PET imaging was correlated with the results of autoradiography. In the operation group, HIF-1a and pimonidazole expression was also noticed in the fundus, whereas HIF-1a and pimonidazole were not expressed in the greater curvature. These results suggest that 64Cu-ATSM PET imaging can detect ischemia of the gastric conduit after devascularization. Hypoxia imaging has been used in several fields, mainly oncology and imaging of stroke patients. In oncology, the ability to determine the degree and extent of tumor hypoxia is important both prognostically and to select patients requiring hypoxia-directed therapies. Also, hypoxia PET imaging can be used in stroke victims to distinguish severely hypoxic viable tissue from reperfused or necrotic tissue [18]. Our study suggests a new potential application of hypoxia imaging after esophagectomy to detect conduit ischemia, which could be very useful in the perioperative management of patients undergoing esophagectomy.

This study had some limitations. First, we could not study whether conduit ischemia was associated with clinical outcomes such as anastomotic leakage. However, to obtain the image of autoradiography and immunohistochemistry, the rats had to be euthanized on postoperative day 1. In addition, we thought that the ischemia was most severe at postoperative day1 and resolved as blood perfusion gradually recovered. In this experiment, we tested whether hypoxia PET imaging was able to detect ischemia per se as a preliminary experiment. Whether conduit ischemia was associated with anastomotic leakage should be explored in further animal studies with longer follow-up periods. Second, more clinical work is needed to verify whether our findings would be useful in human medicine. Finally, several studies reported that Cu-ATSM has tumor type-specific hypoxia selectivity with raising a question as universial hypoxia tracer [19,20], and they suggested that the avid binding of Cu-ATSM to specific tumors might involve other mechanisms independent of hypoxia. However, 64Cu-ATSM has been accepted as a safe radiopharmaceutical that can be used to obtain high-quality images of tumor hypoxia in human cancers and other non-tumor conditions [15–17, 21].

In conclusion, we showed that 64Cu-ATSM hypoxia PET imaging could detect ischemia of a gastric conduit after devascularization in a rat esophagectomy model. Further animal and clinical studies are needed to verify whether hypoxia imaging could be used toward this end in humans.
